# Comparison of the Structure and Diversity of Root-Associated and Soil Microbial Communities Between Acacia Plantations and Native Tropical Mountain Forests

**DOI:** 10.3389/fmicb.2021.735121

**Published:** 2021-09-29

**Authors:** Kozue Sawada, Shinichi Watanabe, Ho Lam Nguyen, Soh Sugihara, Mayuko Seki, Hana Kobayashi, Koki Toyota, Shinya Funakawa

**Affiliations:** ^1^Graduate School of Global Environmental Studies, Kyoto University, Kyoto, Japan; ^2^University of Agriculture and Forestry, Hue University, Hue, Vietnam; ^3^Institute of Agriculture, Tokyo University of Agriculture and Technology, Tokyo, Japan; ^4^Institute of Symbiotic Science and Technology, Tokyo University of Agriculture and Technology, Tokyo, Japan

**Keywords:** acacia plantations, FUNGuild, microbial diversity, Oxisols, root microbiome, tropical mountain forests, Ultisols

## Abstract

Deforestation of native tropical forests has occurred extensively over several decades. The plantation of fast-growing trees, such as *Acacia* spp., is expanding rapidly in tropical regions, which can contribute to conserve the remaining native tropical forests. To better understand belowground biogeochemical cycles and the sustainable productivity of acacia plantations, we assessed the effects of vegetation (acacia plantations vs. native forests) and soil types (Oxisols vs. Ultisols) on soil properties, including the diversity and community structures of bacteria- and fungi-colonizing surface and subsurface roots and soil in the Central Highlands of Vietnam. The results in surface soil showed that pH was significantly higher in acacia than in native for Oxisols but not for Ultisols, while exchangeable Al was significantly lower in acacia than in native for Ultisols but not for Oxisols. Bacterial alpha diversity (especially within phylum Chloroflexi) was higher in acacia than in native only for Oxisols but not for Ultisols, which was the same statistical result as soil pH but not exchangeable Al. These results suggest that soil pH, but not exchangeable Al, can be the critical factor to determine bacterial diversity. Acacia tree roots supported greater proportions of copiotrophic bacteria, which may support lower contents of soil inorganic N, compared with native tree roots for both Oxisols and Ultisols. Acacia tree roots also supported greater proportions of plant pathogenic *Mycoleptodiscus* sp. but appeared to reduce the abundances and diversity of beneficial ECM fungi compared with native tree roots regardless of soil types. Such changes in fungal community structures may threaten the sustainable productivity of acacia plantations in the future.

## Introduction

Tropical forests play important roles in biogeochemical cycling and global climate regulation, and also are a major reservoir of biodiversity (Lee-Cruz et al., 2013). However, native tropical forests have undergone rapid clearance for agriculture (i.e., deforestation) over the past several decades (Gibbs et al., 2010; Hansen et al., 2013). Recently, extensive areas of non-forest lands are being converted to planted forests (i.e., afforestation and reforestation), and the areas of planted forests occupy approximately 7% of world forests, with the largest proportion in the Asia region (Keenan et al., 2015; Food Agriculture Organization United Nations (FAO), 2020). Plantation of fast-growing trees, such as *Acacia* spp., is expanding rapidly to meet the growing demand for wood (Chaudhary et al., 2016). The high productivity of acacia trees to produce large amounts of wood fiber for paper industries and charcoal for steel industries over a relatively short period can help to reduce the deforestation pressure on remaining native forests (Cossalter and Pye-Smith, 2003). However, uncertainties remain about the sustainability of productivity of acacia plantations (Hung et al., 2016).

Forest conversion such as deforestation, afforestation, and reforestation can significantly affect the belowground biogeochemical cycling, including mineral weathering and organic matter decomposition, through changes in bacterial and fungal community structures (Uroz et al., 2016; Baldrian, 2017). The changes in microbial communities in surface horizons can occur through both changes in the soil physicochemical properties (especially soil pH) and the vegetation species (Schneider et al., 2015; Kerfahi et al., 2016; Brinkmann et al., 2019), although the effects of such changes in subsurface horizons remain unknown (Stone et al., 2014, 2015; Pereira et al., 2017).

Deforestation with burning initially raises soil pH by adding base-rich ash from combusted tree biomass, and the effect lasts for decades (McGrath et al., 2001). In contrast, according to a global meta-analysis, afforestation and reforestation typically decreases soil pH by increased uptake of cations by planted trees (Berthrong et al., 2009). Soil pH is widely recognized as the most important factor affecting the structure and diversity of belowground microbial communities after land use changes (Zhou et al., 2020), and bacterial alpha diversity has often decreased with decreasing soil pH in tropical regions (Tripathi et al., 2012, 2016). In addition, exchangeable Al (exch. Al) increases in acidic soil below a pH of 5.5 and is known as a toxic element for plant roots and microbial activities (Foy, 1984; Kunito et al., 2016; Jones et al., 2019). However, the effect of exch. Al on the structure and diversity of microbial communities was rarely investigated. Only Lammel et al. (2018) statistically found that soil pH and exch. Al differently affected each taxon of soil bacterial communities in tropical agricultural soils with pH from 4 to 6.

Tree species compositions in forest vegetation can also affect the belowground microbial communities (Prescott and Grayston, 2013; Liu et al., 2018), in particular, root-associated communities because specific tree roots are typically associated with specific groups of microbes, although soil properties often have influence on root-associated communities (Bonito et al., 2014; Goldmann et al., 2016; Ballauff et al., 2021). The roots of most trees are associated with either arbuscular mycorrhizal fungi or ectomycorrhizal (ECM) fungi (Smith and Read, 2010). In addition, Sawada et al. (2021) observed that roots in natural forest supported greater proportions of copiotrophic plant-growth-promoting bacteria such as Bradyrhizobiaceae and *Burkholderia* sp. compared with roots in a cedar plantation in a temperate region. Brinkmann et al. (2019) observed the increases in pathogenic fungi in monoculture plantations compared with native tropical rain forests in Sumatra, Indonesia. Since mycorrhizal fungi and plant-growth-promoting bacteria may improve tree productivity, while plant pathogen may reduce it, examining root-associated microbial communities can extend our understanding not only on belowground biogeochemical cycles but also on tree productivity and sustainability in forest ecosystems.

In Vietnam, forest cover has changed dramatically from 57.0% in 1950 and steadily declined over the next 40 years to 28.3% in 1990 (Food Agriculture Organization United Nations (FAO), 2015) due to widespread herbicide use during the Indochina wars and unsustainable logging and land-use practices (Cochard et al., 2017; Do et al., 2019). Afterward, Vietnam has experienced a forest transition from net deforestation to net reforestation (Mather, 2007), and its forest cover has increased to 40.4% in 2010 (Food Agriculture Organization United Nations (FAO), 2015). Vietnam has over 400,000 ha of acacia plantations, including over 220,000 ha of clonal *Acacia* hybrid (*Acacia mangium* × *Acacia auriculiformis*) (Sein and Mitlöhner, 2011; Dong et al., 2014).

In the Central Highlands of Vietnam, Oxisols are widely distributed on mafic rocks as parent materials, which are rare in Southeast Asia (United States Department of Agriculture, 2015), while Ultisols are slightly distributed on felsic rocks (Watanabe et al., 2017). This area is originally covered by tropical mountain forests with evergreen broadleaf trees. Deforestation in this area started later than in other regions of Vietnam from 1990 mainly due to cash crop expansion such as coffee and rubber (Cochard et al., 2017). Only recently, many cash crop fields have been combusted and converted to acacia plantations, in which the effect of adding base-rich ash lasts until now. The effect of ash on soil pH and exch. Al should be different between Oxisols and Ultisols, since Oxisols typically contain kaolin minerals and Fe and Al oxides with lower capacities to buffer soil pH, while Ultisols contain weatherable 2:1 clay minerals with relatively higher buffering capacities (Shibata et al., 2017). Therefore, the soil and root samples corrected from both newly occurred acacia plantations and native forests in both Oxisols and Ultisols in this region are the ideal materials to investigate the effects of tree species compositions and soil properties including soil pH and exch. Al on belowground microbial communities.

To better understand not only belowground biogeochemical cycles but also the sustainable productivity of acacia plantations, we investigated the effect of vegetation (acacia plantations vs. native forests) and soil types (Oxisols vs. Ultisols) on the diversity and structure of root-associated and soil bacterial and fungal communities in surface and subsurface horizons using a high-throughput amplicon sequencing technique. We hypothesized that: (1) the vegetation would affect root-associated microbial communities regardless of soil types, and (2) the vegetation would differently affect soil microbial communities between Oxisols and Ultisols through the different changes in soil pH and exch. Al by adding ash, especially in surface soil.

## Materials and Methods

### Study Site and Sampling

The study sites were located in So’ n Lang commune, K’Bang district, Gia Lai province, in the northern part of the Central Highlands region of Vietnam (N14°21′, E108°34′; 780–900 m a.s.l.), where the mean annual temperature is 22.0°C, and the mean annual precipitation is 1,864 mm.

Our sampling sites were acacia plantations and native forests in Oxisols and Ultisols, a total of four sites within approximately 7 km, under similar climatic and topographic conditions. In this region, intact native forests are now very limited. The main species in the native forests are *Lithocarpus* sp., *Dialium cochinchinense*, *Machilus* spp., *Syzygium jambos*, etc., and their compositions differ slightly between Oxisols and Ultisols: being higher in *Lithocarpus* sp. in Oxisols and *Dialium cochinchinense* in Ultisols. The acacia plantations in our sampling sites have been planted with *Acacia* hybrid since 2015 and 2014 in Oxisols and Ultisols after combusting coffee trees, which were planted since 1990, like most of the acacia plantations in this region. Aboveground biomasses of acacia plantations in autumn 2018, calculated by the tree allometric equation using mean diameters at breast height (Hiratsuka et al., 2010), were 74.3 and 50.5 Mg ha^–1^ for Oxisols and Ultisols, which were within the range of growth rates for *Acacia* hybrid in Vietnam (Hung et al., 2016).

Soil and root samples were collected from each four sites in autumn 2018. Three sampling plots in each site were prescribed as three field replicates, which were ca. 10 m apart. In each sampling plot, a soil pit with 1-m wide and 40-cm depth was dug, and mineral surface (0–5 cm) and subsurface (25–30 cm) soil samples were taken from three points within each horizon by a garden trowel to form composite samples with a total amount of about 1 kg. Surface (0–5 cm) and subsurface (25–30 cm) soil samples were within A and B horizons, respectively, in all sampling plots (*n* = 12), and then, the root samples were separated randomly regardless of root morphology from the soil samples in the field. All samples were kept at about 4°C following sampling and during transport for about 2 weeks after sampling. Immediately after arriving at the Japanese laboratory, all soil samples were sieved through a 2-mm mesh. Soil subsamples were air dried for chemical analyses, and the remaining soil subsamples and roots were stored at field moisture at −25°C until use for DNA extraction.

### Soil Chemical Analyses

Soil pH was determined in a 1:5 water-soluble extract. Exchangeable Al^3+^ (exch. Al) was determined by an atomic absorption spectrophotometer (ZA3300, Hitachi, Japan) after 1 M KCl extraction. Total C and N were measured using a dry combustion method with an NC analyzer (SUMIGRAPH NC-TR-22, Sumika Chemical Analysis Service, Ltd., Japan). Inorganic N (NH_4_^+^ and NO_3_^–^) was extracted from 2 g of soil (dry base) with 10 ml of 1 M KCl for 30 min on a shaker, and the suspension was filtered through filter paper (No. 5C, Advantec, Co., Ltd, Japan). NH_4_^+^–N in the extract was analyzed using the modified indophenol blue method (Rhine et al., 1998) with a spectrophotometer (UV-1280, Shimadzu, Co., Ltd., Japan). NO_3_^–^–N in the extract was analyzed by flow injection analysis using a flow-through visible spectrophotometer (S3250, Soma Optics, Ltd., Japan). All soil measurements were expressed on an oven-dry soil weight basis (105°C, 24 h).

### DNA Extraction and Quantitative PCR

Total soil DNA was extracted from 500 mg of each soil sample using ISOIL for Beads Beating kit (Nippon Gene Co., Ltd., Tokyo, Japan) following the protocol of the manufacturer. For root DNA extraction, root tissue up to 1 cm from the root tip was separated from root sample using a sterile pair of scissors after washing the roots three times with sterile deionized water in order to remove adhering soil particles. Total root DNA was extracted from the 30 roots tips with two laboratory replicates using ISOIL (Nippon Gene Co., Ltd., Tokyo, Japan) without bead beating to avoid extracting root DNA (Toju and Sato, 2018). The DNA was eluted in 100 μl of TE buffer.

Bacterial and fungal gene copy numbers were quantified by real-time SYBR Green PCR assays in a StepOne Real-Time PCR System (Life Technologies Japan, Tokyo, Japan) with the 16S rRNA gene primer pair Eub338 and Eub518 and the 18S rRNA gene primer pair 5.8 s and ITS1f (Rousk et al., 2010) as previously described (Sawada et al., 2021). Standard curves were obtained using a 10-fold serial dilution of a plasmid containing either the *Escherichia coli* 16S rRNA gene or the *Saccharomyces cerevisiae* 18S rRNA gene. The measurements were expressed as copy numbers on an oven-dry soil weight basis (105°C, 24 h).

### PCR and Amplicon Sequencing

The amplicon sequencing was performed as previously described (Sawada et al., 2021) with minor modifications. Briefly, the bacterial 16S rRNA gene was amplified using the primer pair 515F (5′-GTGCCAGCMGCCGCGGTAA-3′) and 806R (5′-GGACTACHVGGGTWTCTAAT-3′) (Caporaso et al., 2011). The fungal ITS region was amplified using the primer pair ITS1F_KYO1 (5′-CTHGGTCATTTAGAGGAASTAA-3′) and ITS2_KYO2 (5′-TTYRCTRCGTTCTTCATC-3′) (Toju et al., 2012). For fungi, we pooled two laboratory replicates of DNA extracted from root samples to one to obtain enough DNA contents for amplicon sequencing. The first PCR reactions were performed using the pairs of primers without tags to obtain enough amount of DNA to amplify directly with tagged pairs of primers. Then, 2 μl of each PCR product was used as DNA insert for a second PCR reaction with 10 cycles performed with tagged pair of primers as recommended by Illumina as well as 8-bp tags specific for each sample. Each PCR amplicon was cleaned using an Agencourt AMpure XP system (Beckman Coulter, Brea, CA, United States) to remove short DNA fragments. The quantities and length of the PCR products was verified by Qubit and Fragment Analyzer instrument (Advanced Analytical). The amplicons were mixed and sequenced on an Illumina MiSeq sequencing system (Illumina, San Diego, CA, United States) using the MiSeq Reagent Kit v2.

### Bioinformatics and Analysis of Microbial Community Structures

After quality filtering with a minimum Sanger quality of 20 and a minimum length of 40, the sequencing data were analyzed by QIIME (Caporaso et al., 2010) with default parameters. Chimeric sequences were identified and removed by UCHIME algorithm (Edgar et al., 2011). The remaining sequences were clustered into operational taxonomic units (OTUs) based on 97% identity threshold. Phylogenetic assignation was performed on a consensus sequence from each OTU using the Greengene and UNITE database for bacteria and fungi, respectively. For bacteria, chloroplast, mitochondria, and low-abundance OTUs represented by five or fewer sequences in all samples were removed for the following analysis. For fungi, singleton OTUs in all samples were removed. Furthermore, we classified fungal OTUs into functional groups using the program FUNGuild 1.1 (Nguyen et al., 2016). We only focused on taxa with confidence levels “highly probable” or “probable.” We picked the ECM and plant pathogen guilds.

### Statistics

To calculate the bacterial and fungal diversity metrics, a subset of 20,000 and 5,000 sequences from each sample dataset for bacteria and fungi, respectively, was randomly selected to avoid potential bias caused by sequencing depth. The richness and diversity within each sample (alpha diversity) were estimated using observed number of OTUs and Shannon diversity index. The difference of microbial community structures among samples (beta diversity) was represented on a non-metric multidimensional scaling (NMDS) analyses on a Bray–Curtis dissimilarity matrix between samples. Permutational multivariate analysis of variance (PERMANOVA) was performed with 10,000 permutations to test the effect of horizons (surface vs. subsurface), compartments (roots vs. soil), vegetation (acacia vs. native), and soil types (Oxisols vs. Ultisols) on microbial community structures. A heat map for the hierarchical clustering of more than 1% of the average relative abundances at phylum–class level and at family level was generated using Morisita–Horn dissimilarity matrix between samples. The analyses were conducted using the package “vegan” (Oksanen et al., 2013) of the R v.3.3.0 project (R Core Team, 2016).

The effects of vegetation and soil types were tested by two-way ANOVA analyses and a *post hoc* Tukey’s test only if an interactive effect was significant, after dividing the data into four groups of surface roots, subsurface roots, surface soil, and subsurface soil. The relative abundance values at phylum–class and at family levels at more than 1% of the average abundance and at genus level within ECM and plant pathogen guilds were compared after transformation to arcsine square root to achieve a normal distribution.

## Results

### Soil Biochemical Properties

In surface soil, a significantly higher pH of mean 0.8 U was measured in acacia than in native for Oxisols, but no difference was observed for Ultisols (Table 1). In contrast, exch. Al in Acacia was significantly lower than in native for Ultisols, but no difference was observed for Oxisols (Table 1). In subsurface soil, although exch. Al was significantly higher in native than in acacia, and for Ultisols than for Oxisols, the ranges of soil pH and exch. Al among the four sites were narrow compared with surface soil.

**TABLE 1:** Soil biochemical properties in surface and subsurface horizons in two vegetations (Acacia and Native; V) for two soil types (Oxisols and Ultisols; S).

		Oxisols		Ultisols				
		Acacia	Native	Acacia	Native	V	S	V × S
pH	Surface	5.1 (0.1)^a^	4.3 (0.1)^c^	4.9 (0.0)^ab^	4.8 (0.1)^b^	***	*	***
	Subsurface	5.1 (0.0)^a^	4.9 (0.1)^ab^	4.8 (0.1)^b^	4.9 (0.0)^ab^	NS	*	*
Exchangeable Al (cmol_*c*_ kg^–1^)	Surface	1.9 (0.2)^bc^	2.5 (0.5)^ab^	1.2 (0.5)^c^	3.1 (0.4)^a^	***	NS	*
	Subsurface	0.5 (0.1)	1.2 (0.2)	1.2 (0.0)	1.8 (0.1)	***	***	NS
Total C (mg g^–1^)	Surface	50 (8)	43 (7)	11 (1)	17 (3)	NS	***	NS
	Subsurface	19 (3)	18 (3)	8 (1)	7 (1)	NS	***	NS
Total N (mg g^–1^)	Surface	3.0 (0.2)	3.4 (0.2)	1.0 (0.1)	1.7 (0.0)	***	***	NS
	Subsurface	1.3 (0.1)	1.4 (0.1)	0.6 (0.1)	0.7 (0.1)	NS	***	NS
NH_4_^+^-N (μg g^–1^)	Surface	56.6 (7.7)	93.4 (11.4)	33.1 (7.1)	76.1 (3.0)	***	**	NS
	Subsurface	32.4 (3.6)	46.8 (2.1)	18.9 (4.4)	31.1 (4.5)	***	***	NS
NO_3_^–^-N (μg g^–1^)	Surface	22.8 (2.4)	28.4 (6.6)	7.6 (3.2)	7.8 (1.0)	NS	***	NS
	Subsurface	4.1 (0.6)^b^	6.1 (0.3)^a^	2.8 (1.3)^bc^	1.7 (0.1)^c^	NS	***	**
Bacteria (copies *10^9^ g^–1^)	Surface	235 (18)	316 (52)	57 (14)	91 (28)	*	**	NS
	Subsurface	127 (106)	147 (45)	48 (21)	42 (18)	NS	**	NS
Fungi (copies *10^9^ g^–1^)	Surface	29 (15)	27 (9)	4 (1)	6 (4)	NS	***	NS
	Subsurface	1.4 (0.8)	2.5 (0.7)	0.4 (0.4)	0.5 (0.4)	NS	*	NS

*Data are means (standard deviations) of three replicates.*

NS, not significant; *P < 0.05, **P < 0.01, and ***P < 0.001, respectively, by two-way ANOVA.

*Different letters indicate significant differences (P < 0.05) by multiple comparison if an interactive effect was significant.*

In both surface and subsurface soils, total C and N, inorganic N, and bacterial and fungal gene copies were significantly higher for Oxisols than for Ultisols (Table 1). NH_4_^+^–N in surface and subsurface soils, and total N and bacterial gene copies in surface soil, were significantly lower in acacia than in native (Table 1).

### Alpha and Beta Diversity Patterns

**FIGURE 1:**
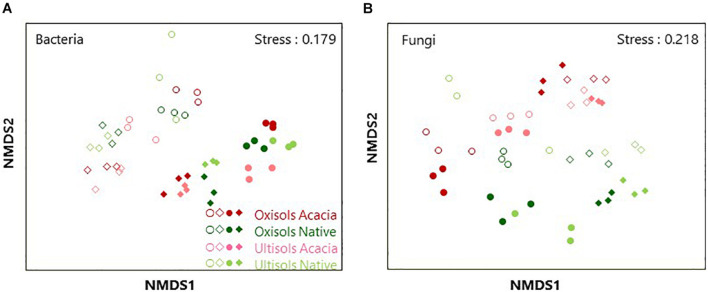
Non-metric multidimensional scaling (NMDS) ordination using Bray-Curtis dissimilarity index of **(A)** bacterial and **(B)** fungal OTU distribution for surface root (blank squares), subsurface root (blank circles), surface soil (filled squares) and subsurface soil (filled circles).

We assessed a total of 2,858,557 and 1,253,527 sequences (average 39,702 and 26,115 per sample) for bacteria and fungi, respectively. Both bacterial and fungal community structures were clustered in the NMDS ordination (Figure 1). PERMANOVA showed that horizons (*R*^2^ = 0.106, *p* < 0.001 and *R*^2^ = 0.081, *p* < 0.001), compartments (*R*^2^ = 0.296, *p* < 0.001 and *R*^2^ = 0.080, *p* < 0.001), vegetation (*R*^2^ = 0.074, *p* < 0.01 and *R*^2^ = 0.063, *p* < 0.001), and soil types (*R*^2^ = 0.049, *p* < 0.05 and *R*^2^ = 0.060, *p* < 0.001) significantly influenced both bacterial and fungal community structures, respectively. Since horizons and compartments more affected the microbial community structures than vegetation and soil types, the effect of vegetation and soil types was tested after dividing the data into surface roots, subsurface roots, surface soil, and subsurface soil to avoid interactive effects of horizons or compartments. As a result, vegetation more significantly influenced both bacterial and fungal community structures than soil types in surface roots and soil (Table 2), while soil types more affected those than vegetation in subsurface soil (Table 2). Regarding the subsurface roots, bacterial communities differed significantly between vegetation (*p* < 0.01) and soil types (*p* < 0.05), while fungal communities differed significantly between soil types (*p* < 0.01) but not vegetation (Table 2).

**TABLE 2:** Effects of vegetations (Acacia and Native) and soil types (Oxisols and Ultisols) on bacterial and fungal community structures in surface and subsurface roots and soils.

	Vegetation		Soil types	
	*R* ^2^	*P*	*R* ^2^	*P*
**Surface roots**				

Bacteria	0.355	0.002**	0.113	0.204^*NS*^
Fungi	0.244	0.003**	0.096	0.300^*NS*^

**Subsurface roots**				

Bacteria	0.218	0.002**	0.164	0.039*
Fungi	0.143	0.083^*NS*^	0.191	0.014*

**Surface soil**				

Bacteria	0.371	0.003**	0.233	0.020*
Fungi	0.217	0.003**	0.149	0.039*

**Subsurface soil**				

Bacteria	0.195	0.033*	0.334	0.002**
Fungi	0.141	0.040*	0.199	0.003**

NS, not significant; *P < 0.05, and **P < 0.01, respectively, by PERMANOVA.

Bacterial and fungal OTU richness and Shannon diversity indices for surface and subsurface roots were not significantly different between vegetations and between soil types, except for fungal OTU richness in surface roots, being higher in native than in acacia only for Ultisols (Table 3). Bacterial OTU richness and Shannon diversity for surface soil was significantly higher in Acacia than in native for Oxisols but not for Ultisols (Table 3), which was the same statistical result as soil pH but not exch. Al (Table 1).

**TABLE 3:** Richness and diversity index values of the bacterial and fungal communities in two vegetations (Acacia and Native; V) for two soil types (Oxisols and Ultisols; S).

	Oxisols		Ultisols				
	Acacia	Native	Acacia	Native	V	S	V × S
**Surface roots**							

Bacterial OTU richness	1852 (171)	1857 (158)	1917 (145)	2156 (23)	NS	NS	NS
Bacterial Shannon index	5.22 (0.33)	5.50 (0.34)	5.51 (0.18)	5.59 (0.10)	NS	NS	NS
Fungal OTU richness	159 (38)^ab^	111 (7)^b^	126 (13)^b^	205 (18)^a^	NS	*	**
Fungal Shannon index	3.11 (0.36)	3.02 (0.43)	3.08 (0.45)	3.58 (0.19)	NS	NS	NS

**Subsurface roots**							

Bacterial OTU richness	1450 (144)	1605 (67)	1618 (69)	1399 (490)	NS	NS	NS
Bacterial Shannon index	5.13 (0.16)	5.43 (0.06)	5.34 (0.28)	5.14 (0.47)	NS	NS	NS
Fungal OTU richness	40 (18)	44 (2)	53 (8)	45 (38)	NS	NS	NS
Fungal Shannon index	2.38 (0.63)	2.06 (0.56)	1.86 (0.65)	2.36 (0.63)	NS	NS	NS

**Surface soil**							

Bacterial OTU richness	1988 (61)^a^	1644 (92)^b^	1733 (161)^ab^	1784 (134)^ab^	NS	NS	*
Bacterial Shannon index	6.12 (0.04)^a^	5.57 (0.02)^c^	6.01 (0.16)^ab^	5.80 (0.04)^b^	***	NS	**
Fungal OTU richness	140 (33)	176 (24)	158 (10)	176 (12)	NS	NS	NS
Fungal Shannon index	2.31 (0.36)	3.11 (0.48)	3.55 (0.42)	3.67 (0.41)	NS	*	NS

**Subsurface soil**							

Bacterial OTU richness	1439 (10)	1485 (91)	1223 (94)	1199 (161)	NS	**	NS
Bacterial Shannon index	5.44 (0.02)^a^	5.52 (0.10)^a^	5.47 (0.12)^a^	5.25 (0.14)^a^	NS	NS	*
Fungal OTU richness	41 (9)	68 (9)	77 (22)	104 (34)	NS	*	NS
Fungal Shannon index	2.28 (0.51)	2.86 (0.10)	2.98 (0.23)	3.33 (0.61)	NS	*	NS

*Data are means (standard deviations) of three replicates.*

NS, not significant; *P < 0.05, **P < 0.01, and ***P < 0.001, respectively, by two-way ANOVA.

*Different letters indicate significant differences (P < 0.05) by multiple comparison if an interactive effect was significant.*

**FIGURE 2:**
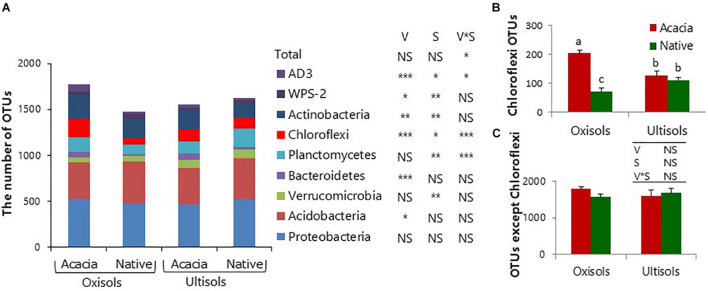
Number of **(A)** total bacterial OTUs, **(B)** Chloroflexi OTUs, and **(C)** OTUs after removing Chloroflexi OTUs for surface soil. Bars show standard deviations of the means. NS, *, **, and *** in inserted tables indicate not significant, *P <* 0.05, *P <* 0.01, and *P <* 0.001, respectively, by two-way ANOVA, V and S indicate Vegetation and Soil types. Different letters indicate significant differences (*P* < 0.05).

We further analyzed OTU richness in each bacterial phylum for surface soil to determine which bacterial phyla mostly contributed to the difference of total bacterial diversity (Figure 2A). The OTU richness in only Chloroflexi showed the same statistical result as total bacterial OTU richness, which was significantly higher in acacia than in native for Oxisols but not for Ultisols (Figure 2B and Table 3). In addition, the numbers after removing the numbers of OTUs in Chloroflexi from the total numbers were not significantly different between vegetation and between soil types (Figure 2C).

### Bacterial Taxonomic Distribution

**FIGURE 3:**
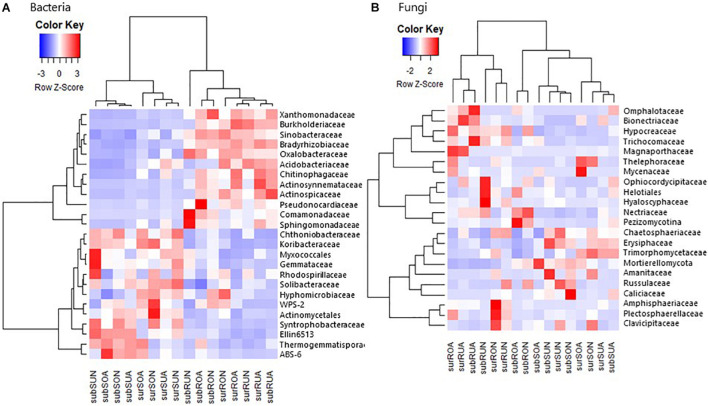
A heat map for the hierarchical clustering of **(A)** bacterial and **(B)** fungal average relative abundances at family level, Taxa with more than 1% of the abundance are represented. Relative abundance of each taxon was transformed into a row Z-score, and darker red and blue indicate higher and lower abundances, respectively, sur, sub, R, S, O, U, A, and N in *x* axis indicate surface, subsurface, Root, Soil, Oxisols, Ultisols, Acacia, and Native, respectively.

We applied heat map analysis with hierarchical clustering to intuitively display the differences in relative abundance of microbial taxa among samples. Hierarchical clustering analysis separated bacterial taxa initially into roots and soil samples both at phylum-class level (Supplementary Figure 2) and at family level (Figure 3). Root samples had high Copiotrophic bacteria such as Alphaproteobacteria (mainly Bradyrhizobiaceae and Sphingomonadaceae, but not Hyphomicrobiaceae and Rhodospirillaceae), Betaproteobacteria (mainly Burkholderiaceae, Comamonadaceae, and Oxalobacteraceae), Gammaproteobacteria (mainly Sinobacteraceae and Xanthomonadaceae), Actinobacteria (mainly Actinospicaceae, Actinosynnemataceae, and Pseudonocardiaceae, but not unidentified Actinomycetales), and Bacteroidetes (mainly Chitinophagaceae) (Figure 3 and Supplementary Figures 1, 2). In contrast, soil samples had high oligotrophic bacteria such as Deltaproteobacteria (mainly unidentified Myxococcales and Syntrophobacteraceae), Acidobacteria (mainly Koribacteraceae, unidentified Ellin6513 and Solibacteraceae, but not Acidobacteriaceae), Verrucomicrobia (mainly Chthoniobacteraceae), Planctomycetes (mainly Gemmataceae), Chloroflexi (mainly Thermogemmatisporaceae), WPS-2, and AD3 (mainly ABS-6) (Figure 3 and Supplementary Figures 1, 2).

**FIGURE 4:**
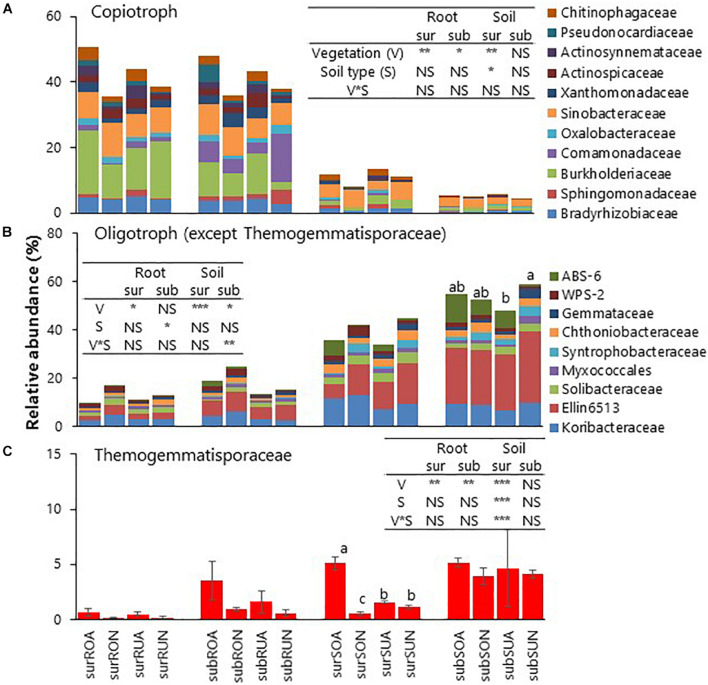
Relative abundances of **(A)** copiotroph, **(B)** Oligotroph (except Thermogemmatisporaceae) and **(C)** Thermogemmatisporaceae. sur, sub, R, S, O, U, A, and N indicate surface, subsurface, Root, Soil, Oxisols, Ultisols, Acacia, and Native, respectively. NS, *, **, and *** in inserted tables indicate not significant, *P* < 0.05, *P* < 0.01, and *P <* 0.001, respectively, by two-way ANOVA. Different letters indicate significant differences (*P* < 0.05).

Relative abundances of total copiotrophic bacteria enriched in roots were significantly greater in acacia than in native for surface and subsurface roots in both Oxisols and Ultisols (Figure 4A). Within copiotrophic bacterial families, Chitinophagaceae was significantly greater in Acacia than in Native, while Bradyrhizobiaceae, Oxalobacteraceae, and Sinobacteraceae were not significantly different between vegetation for surface and subsurface roots (Supplementary Table 3).

Relative abundances of most oligotrophic bacteria such as Syntrophobacteraceae, unidentified Ellin6513, and Solibacteraceae enriched in soil were significantly greater in native than in acacia for surface soil in both Oxisols and Ultisols (Figure 4B and Supplementary Table 3). In contrast, the relative abundance of only oligotrophic Thermogemmatisporaceae (phylum Chloroflexi) for surface soil was significantly greater in acacia than in native for Oxisols but not for Ultisols (Figure 4C and Supplementary Table 3), which was the same statistical result as soil pH (Table 1).

### Fungal Taxonomic Distribution

Fungal taxonomic distributions were initially clustered between roots and soil both at phylum–class level (Supplementary Figure 2) and family level (Figure 3) by clustering heatmap analyses. Sordariomycetes (mainly Hypocreaceae, Nectriaceae, and Magnaporthaceae) appeared to be greater in roots than in soil, while Mortierellomycota appeared to be greater in soil than in roots, although the differences between roots and soil were not clear compared with bacterial taxa (Figure 3 and Supplementary Figures 1, 2).

**FIGURE 5:**
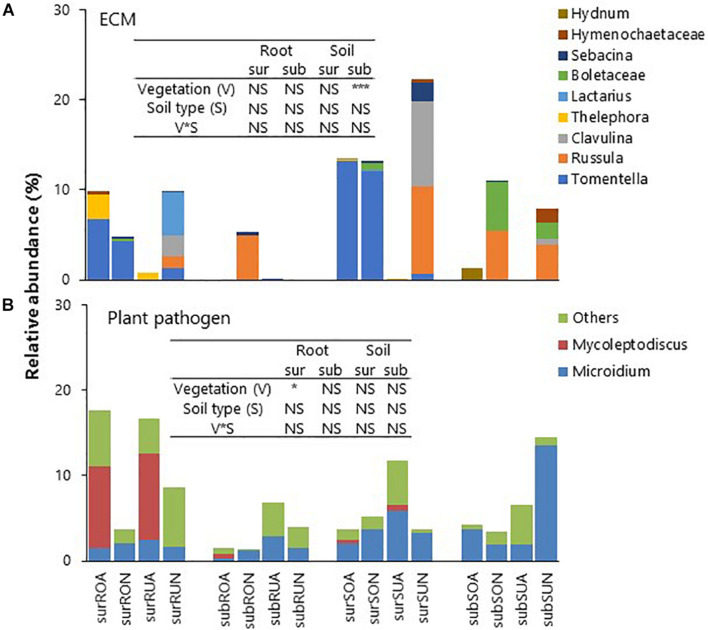
Relative abundances of **(A)** ECM and **(B)** Plant pathogen, sur, sub, R, S, O, U, A, and N indicate surface, subsurface, Root, Soil, Oxisols, Ultisols, Acacia, and Native, respectively. NS, *, and *** in inserted tables indicate not significant, *P* < 0.05 and *P* < 0.001, respectively, by two-way ANOVA.

Relative abundances of ECM taxa classified by FUNGuild were significantly greater in native than in acacia for subsurface soil in both Oxisols and Ultisols (Figure 5A). The dominant ECM taxa in native forests were the *Tomentella* spp., *Russula* spp., *Clavulina* sp., and Boletaceae. The ECM (mainly *Tomentella* spp.) were detected for Oxisol surface roots and soil in acacia (Figure 5A). Relative abundances of plant pathogens (mainly *Mycoleptodiscus* sp.) classified by FUNGuild were significantly greater in acacia than in native for surface roots in both Oxisols and Ultisols (Figure 5B).

## Discussion

### Effects of Vegetation and Soil Types on Soil pH and Exchangeable Aluminum

We found that the effect of vegetation on pH and exch. Al in surface soil was different between Oxisols and Ultisols (Table 1). In the native forests, when higher root activities and higher rates of nutrient cycles accelerate acidification in surface compared with subsurface soil, highly weathered Oxisols with a lower buffering capacity can decrease soil pH, while Ultisols with a relatively higher buffering capacity can suppress soil pH decrement by solubilizing Al (Shibata et al., 2017). Thus, soil pH decrement in surface compared with subsurface in native forests was greater for Oxisols than for Ultisols. In acacia plantations, the addition of base cations from combusted trees to surface soil directly increased soil pH for Oxisols, but precipitated Al to buffer soil pH for Ultisols. Therefore, acid neutralization by adding base cations would more easily increase the pH in surface Oxisols and would more easily decrease the exch. Al in surface Ultisols (Table 1).

### Effects of Vegetation on Microbial Community Structures

We found that vegetation, but not soil types, strongly influenced root-associated bacterial and fungal community structures in the surface based on the PERMANOVA analyses in our study sites (Table 2), although there were several reports that soil types affected root-associated microbial communities (Bonito et al., 2014; Ballauff et al., 2021). Therefore, tree roots appeared to mainly control root-associated microbial communities regardless of soil types in this region (Table 2), supporting our first hypothesis in surface.

On bacterial taxonomic distribution, it was reasonable that the relative abundances of copiotrophic bacteria such as Alpha-, Beta-, and Gamma-proteobacteria and Bacteroidetes (Eilers et al., 2010) were greater in roots than in soil (Figure 3 and Supplementary Figures 1, 2), since tree roots exudate labile C compounds, such as sugars, amino acids, and organic acids (Uroz et al., 2016; Lladó et al., 2017). Intriguingly, the relative abundances of total copiotrophic bacteria enriched in roots were significantly greater in acacia than in native for both surface and subsurface roots regardless of soil types (Figure 4A), suggesting that acacia tree roots more actively selected copiotrophic bacterial taxa. Among copiotrophic bacteria, Bradyrhizobiaceae, which may include N-fixing rhizobia associated with *Acacia* spp. (Le Roux et al., 2009), was not significantly different between vegetation (Supplementary Table 3). Pereira et al. (2017) also found no increases in N-fixing rhizobia in acacia soils compared with that in the Eucalyptus plantation in Brazil. In contrast, Chitinophagaceae, which is known to be a rapid user of root exudates (el Zahar Haichar et al., 2008), was significantly greater in acacia than in native for both surface and subsurface roots (Supplementary Table 3), suggesting that the acacia tree roots appeared to exudate more labile substrates to surrounding roots compared with roots in native forests. It was known that fast-growing plants can invest more of their assimilated-C into root exudation compared with slow-growing plants in grassland ecosystems to accelerate soil N cycling (Kaštovská et al., 2015; Guyonnet et al., 2018). Therefore, fast-growing acacia trees may also adopt the strategy to invest more C to root exudation to acquire more N, which may support lower NH_4_^+^–N in acacia than in native regardless of soil type (Table 1).

The relative abundances of most oligotrophic bacteria, in particular, Acidobacteria including unidentified Ellin6513 and Solibacteraceae, were significantly greater in native than in acacia in surface soil (Figure 4B). The results would be due to soil acidity such as lower soil pH for Oxisols and higher exch. Al for Ultisols in native than in acacia in surface soil, since it is well known that Acidobacteria are greater in acidic soils (e.g., Rousk et al., 2010). In contrast, we found that only oligotrophic Thermogemmatisporaceae involving the phylum Chloroflexi had different trends of other oligotrophic bacteria (Figure 4C), being higher in acacia than in native only for Oxisols but not for Ultisols. More details on Chloroflexi are discussed in the next section.

On fungal taxonomic distribution, relative abundances of well-known ECM fungi such as *Tomentella* spp., *Russula* spp., *Clavulina* sp., and Boletaceae in the native forest soils were about 5–20%, which were comparable with the observations in the other native tropical forest soils (Tripathi et al., 2016; Brinkmann et al., 2019). This is because the native vegetation species consist of ECM-associated trees such as *Lithocarpus* sp. and *Syzygium jambos*. Acacia trees in Oxisols were also associated with ECM *Tomentella* spp., which was known to associate with *Acacia* spp. (Lee et al., 2006). Overall, the trees in the native forests might acquire better nutrients via ECM pathways than acacia trees, although the abundances of ECM taxa were highly variable between sites and even within field replicates due to high spatial heterogeneity of forest ecosystems (Baldrian, 2017).

Plant pathogens classified by the FUNGuild were abundant in acacia surface roots regardless of soil type, although the acacia trees had been growing there only for 3–4 years (Figure 5B). An increase in pathogens in monocultures is a well-known phenomenon (McDonald and Stukenbrock, 2016), for example, Brinkmann et al. (2019) observed that the abundances of pathogens in rubber and oil palm plantations were greater than in those of native tropical rain forests in Sumatra, Indonesia. *Mycoleptodiscus* sp., which is known to be pathogenic to Fabaceae (Hernández-Restrepo et al., 2019), was detected in acacia (Fabaceae) surface roots and soil for both Oxisols and Ultisols (Figure 5B). Until now, no obvious disease symptoms are observed in this region, and there is no report of such species as pathogens to *Acacia* hybrid. However, our results suggest that acacia plantations can foster the proliferation of the plant pathogen, which has a potential risk of disease and, thus, might threaten the sustainability of their productivity.

### Effects of Vegetation and Soil Types on Alpha Diversity

We found that the conversion of the native forests to the acacia plantations had little effect on fungal alpha diversity in surface and subsurface roots and soil (Table 3), while it largely affected fungal community structures (Table 2), in agreement with previous studies in tropical land-use systems (Kerfahi et al., 2016; Brinkmann et al., 2019; Ballauff et al., 2021).

In contrast to fungi, we found that vegetation differently affected bacterial alpha diversity between Oxisols and Ultisols with different capacities to buffer soil pH in surface soil (Table 3), supporting our second hypothesis. In addition, we clearly showed that the increase in soil pH, but not the decrease in exch. Al, significantly increased soil bacterial alpha diversity (Tables 1, 3). The result suggests that forest conversion in Oxisols would more easily affect bacterial diversity compared with Ultisols in surface soils, since adding ash by deforestation and acidification by afforestation and reforestation would more easily affect the pH in Oxisols with lower buffering capacities.

The numbers of OTUs in only Chloroflexi showed the same statistical result as soil pH and bacterial alpha diversity (Figure 2B), and there was no significant effect of vegetation and soil types after removing the numbers of OTUs in Chloroflexi from total numbers (Figure 2C). The results suggested that the diversity of Chloroflexi appeared to mainly contribute to the total bacterial diversity in surface soil. The relative abundances of Thermogemmatisporaceae, which occupy 48% of Chloroflexi, also showed the same statistical result as the bacterial alpha diversity and soil pH (Figure 4C and Supplementary Table 3). The result was consistent with several studies that observed that the relative abundances of Chloroflexi were positively correlated with soil pH among different vegetation types (Tripathi et al., 2016; Deng et al., 2018) and that calcium carbonate application increased the relative abundances of Chloroflexi as well as soil pH and bacterial alpha diversity (Guo et al., 2019). Therefore, Chloroflexi (mainly Thermogemmatisporaceae) appeared to be the key to determine the structures and diversity of bacterial communities through pH changes but not exch. Al by forest conversion in surface soil. The members of the family Thermogemmatisporaceae have a mycelia-forming morphology and the capacity to produce several secondary metabolites like Actinomycetes (Cavaletti et al., 2006). Lammel et al. (2018) observed that some Actinobacteria were more affected by the pH changes than the indirect pH effects like availability of Al. Therefore, the bacteria with such a unique morphology and capacity might directly respond to the pH change by forest conversion, although further study is needed to fully reveal their physiological properties and their roles in soil environments (Yabe et al., 2017).

## Conclusion

Our study revealed two important findings in this region: (1) Acacia tree roots supported greater proportions of copiotrophic bacteria and plant pathogenic fungi (i.e., *Mycoleptodiscus* sp.) but appeared to reduce the abundance and diversity of beneficial ECM fungi compared with the native tree roots, irrespective of soil types. (2) Conversion from the native forests to the acacia plantations increased bacterial alpha diversity (mainly within phylum Chloroflexi) in surface soil through the increase in pH only for Oxisols but not for Ultisols. The increase in plant pathogens and the decrease in ECM fungi may threaten the sustainable productivity of acacia plantations in the future, although forest conversion might increase bacterial diversity depending on the soil pH change. Our analysis is limited to clarify the roles of such changes in the structure and diversity of belowground microbial communities (especially phylum Chloroflexi) on soil functions such as mineral weathering and nutrient cycles. Furthermore, our results must be interpreted with caution due to the following limitations of our sampling design: (1) We compared belowground microbial communities only between a single acacia plantation and a single native forest under Oxisols and Ultisols, and thus, an additional study replicated in different locations is needed to separate a spatial effect from the effects of vegetation and soil types. (2) Since acacia plantations had been planted after combusting coffee trees, coffee trees before planting acacia trees might affect the belowground microbial communities. (3) The differences in the compositions, ages, and biomass of the native forest stands between Oxisols and Ultisols might affect the belowground microbial communities. Nevertheless, our study provides important information on the impact of forest conversion on belowground microbial communities and ecosystem functioning in tropical mountain forests, leading to develop effective microbe-based strategies for sustainable management of acacia plantations.

## Data Availability Statement

The datasets generated for this study can be found in the DNA Data Bank of Japan (DDBJ) Sequence Read Archive under accession numbers DRA012318 and DRA012317 for bacteria and fungi, respectively.

## Author Contributions

KS, SS, and SF designed this study. SF and KT supervised this study. KS, SW, HN, and SF carried out the field work and got the field information. KS, MS, and HK carried out the experiments and data analysis. KS wrote the manuscript. SS and KT revised the manuscript. All authors contributed to the article and approved the manuscript.

## Conflict of Interest

The authors declare that the research was conducted in the absence of any commercial or financial relationships that could be construed as a potential conflict of interest.

## Publisher’s Note

All claims expressed in this article are solely those of the authors and do not necessarily represent those of their affiliated organizations, or those of the publisher, the editors and the reviewers. Any product that may be evaluated in this article, or claim that may be made by its manufacturer, is not guaranteed or endorsed by the publisher.
