# Supplementary material for: Comparison of the Structure and Diversity of Root-Associated and Soil Microbial Communities Between Acacia Plantations and Native Tropical Mountain Forests
Source: Front Microbiol. 2021 Sep 29;12:735121. doi: 10.3389/fmicb.2021.735121 (PMC8511777; doi:10.3389/fmicb.2021.735121)
Supplement: Supplementary file 1 [file Data_Sheet_1.pdf]

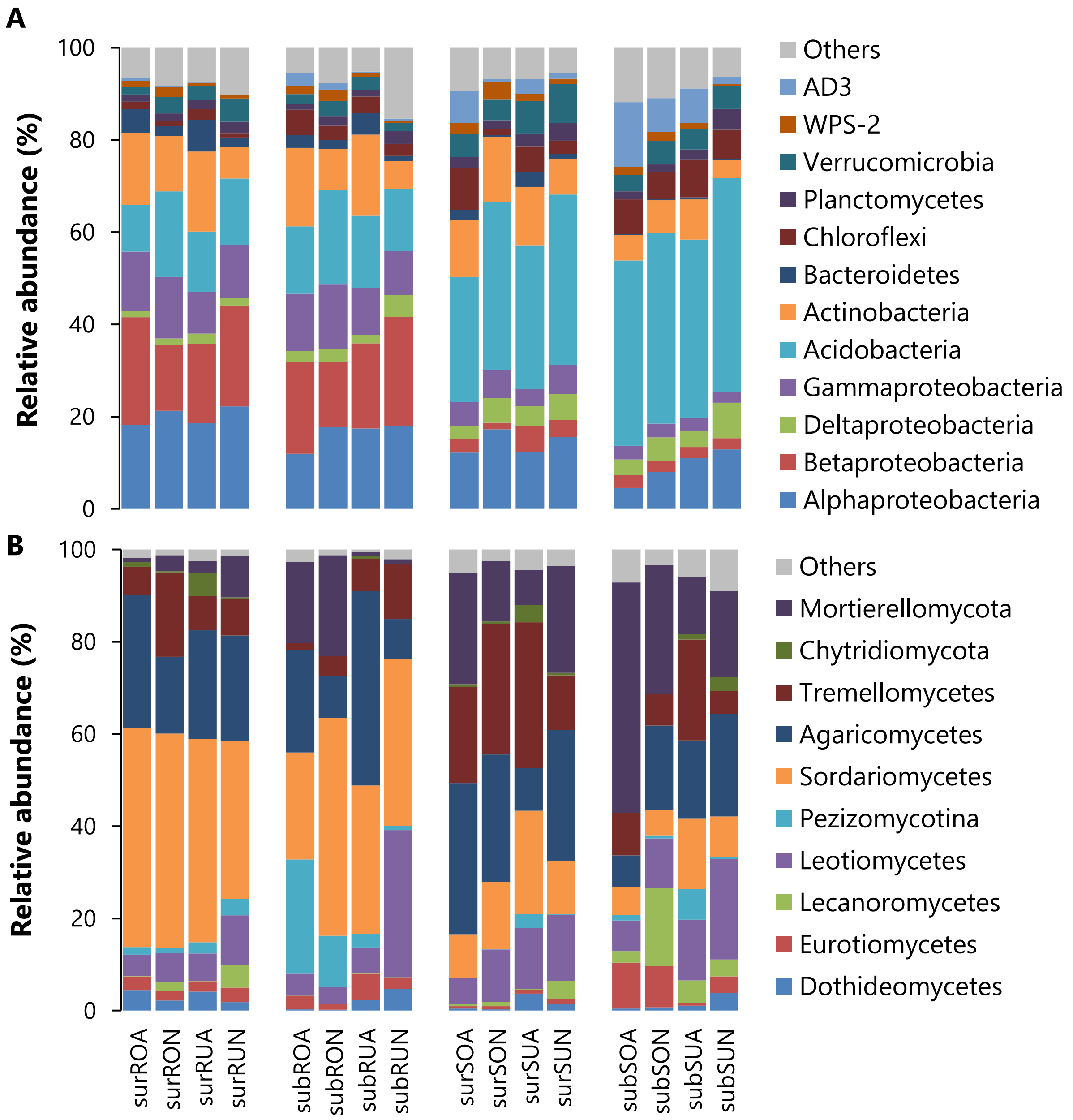

FIGURE S1 | Relative abundances of **(A)** bacterial and **(B)** fungal taxa at the phylum-class level. sur, sub, R, S, O, U, A and N in x axis indicate surface, subsurface, Root, Soil Oxisols, Ultisols, Acacia and Native, respectively.

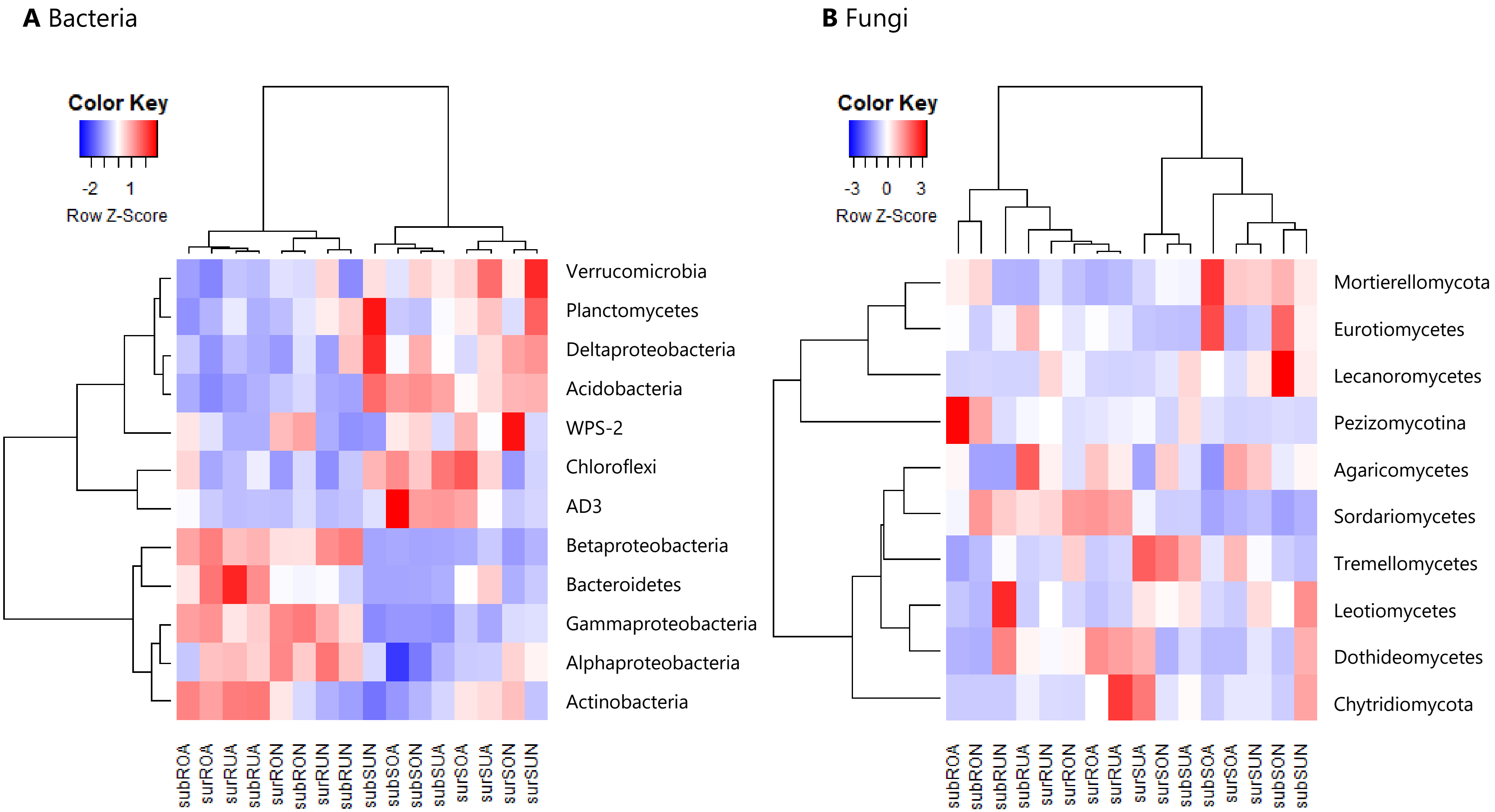

FIGURE S2 | A heat map for the hierarchical clustering of **(A)** bacterial and **(B)** fungal average relative abundances at phylum-class level. Taxa with more than 1% of the abundance are represented. Relative abundance of each taxon was transformed into a row Z-score, and darker red and blue indicate higher and lower abundances, respectively. sur, sub, R, S, O, U, A and N in x axis indicate surface, subsurface, Root, Soil, Oxisols, Ultisols, Acacia and Native, respectively.
